# Dual-energy computed tomography angiography-based quantification of lesion net water uptake to identify stroke onset time

**DOI:** 10.1016/j.heliyon.2023.e23540

**Published:** 2023-12-09

**Authors:** Jiang Jingxuan, Guan Baohui, Zhou Jingyi, Gu Hongmei, Li Minda, Hua Ye, Li Yuehua

**Affiliations:** a Institute of Diagnostic and Interventional Radiology, Shanghai Sixth People's Hospital Affiliated to Shanghai Jiao Tong University School of Medicine, Shanghai, China; b Department of Radiology, Kunshan second People's Hospital, Kunshan, China; c Department of Radiology, Affiliated Hospital of Nantong University, Nantong, China

**Keywords:** Stroke, Symptom onset, Dual-energy, Computed tomography, Net water uptake

## Abstract

**Objectives:**

To explore whether dual-energy computed tomography (DECT) angiography can provide reliable quantitative information on net water uptake (NWU) of ischemic brain to identify stroke patients within 4.5 h.

**Methods:**

We retrospectively reviewed 142 patients with stroke occurrence and who underwent DECT angiography between August 2016 and May 2022. DECT angiography manual drawn the ischemic area by referring to the normal area of the contralateral hemisphere and follow-up images. The NWU in the ischemic area was determined using virtual non-contrast and monoenergetic (VNC &VM) images acquired from DECT angiography. The NWU values in the ischemic area were compared between stroke patients within and beyond 4.5 h. The diagnostic performance of the NWU values derived from the VNC and VM images was assessed through receiver operating characteristic curve analysis. Additionally, Furthermore, we examined the correlation between the NWU values and the stroke onset time.

**Results:**

Seventy-eight (54.93 %) stroke patients underwent DECT angiography and within 4.5 h. These patients with lower median National Institute of Health stroke scale (NIHSS) scores on admission than those beyond 4.5 h (p < 0.05). Furthermore, the group within 4.5 h had lower NWU values than did the group beyond 4.5 h on all VNC and VM images (p < 0.001). The analysis revealed that the NWU values determined using the VM (60 keV) images had the highest predictive efficiency (AUC, 0.95; sensitivity, 100 %; and specificity, 89.06 %) and showed the strongest positive correlation with stroke onset time (r-value = 0.58, p < 0.001).

**Conclusions:**

Our findings showed that DECT angiography-based quantification of NWU helps identify the stroke patients within 4.5 h with high predictive efficiency. Thus, NWU values determined using VM (60 keV) images could serve as a significant biomarker for stroke onset time.

## 1 Introduction

Intravenous thrombolysis can enhance functional outcomes of patients who experience an acute ischemic stroke (AIS) [1]. According to current guidelines, the use of thrombolysis is effective and safe for treating stroke patient within 4.5 h [[2], [3], [4]]. However, the precise time of stroke onset remains unknown in as many as 25 % of patients [5,6]. Fortunately, some unknown stroke time patients can benefit from early treatment based on salvageable brain tissue identified on the mismatch of multimodal computed tomography (CT) or magnetic resonance imaging (MRI) [[7], [8], [9], [10]]. However, the long scan and processing time, motion artifacts, and high price make multimodal advanced series not always feasible after stroke onset.

Cerebral edema is the result of persistent brain hypoxia and the pathophysiological feature of ischemic infarction, which can be monitored by non-contrast CT (NCCT) [11]. The density of NCCT changes in a linear fashion with the percentage change in fractional brain tissue water; thus, net water uptake (NWU) values per brain tissue volume can be calculated [12]. The predictive power of NWU values determined on NCCT for identifying the lesion age of patients with stroke symptom onset was comparable to that of mismatch sign on multimodal advanced series [13,14]. Moreover, CT angiography (CTA) detects hypoattenuation resulting from significant reductions in brain blood volume or prolonged delay of contrast to the infarcted brain not just as cytotoxic edema on NCCT [15]. Compared with NCCT, CTA was found to be a better method for distinguishing cerebral edema caused by ischemic brain tissue and seems more useful and reliable [16,17].

Dual-energy CT (DECT) is a standard clinical technique that enables material characterization with differential CT attenuation at two distinct energy levels [18]. DECT angiography enables the reliable derivation of virtual images [19,20], which means both virtual “NCCT” and “CTA” images can be obtained simultaneously through DECT angiography reconstructions. Several previous findings have reported the value of virtual images reconstructed from DECT to optimize the visualization of acute cerebral infarction, in comparison with that achieved using conventional NCCT [[21], [22], [23]]. However, few reports have focused on the detection ability of DECT angiography in early cerebral edema of stroke. We hypothesized that the NWU values determined using VNC and VM images reconstructed from DECT angiography may provide reliable quantification of NWU in ischemic lesions. Thus, this study was aimed at exploring whether DECT angiography-based NWU can aid in the identification of stroke patients within 4.5 h.

## 2 Materials and methods

### 2.1 Patients

**Fig. 1:**
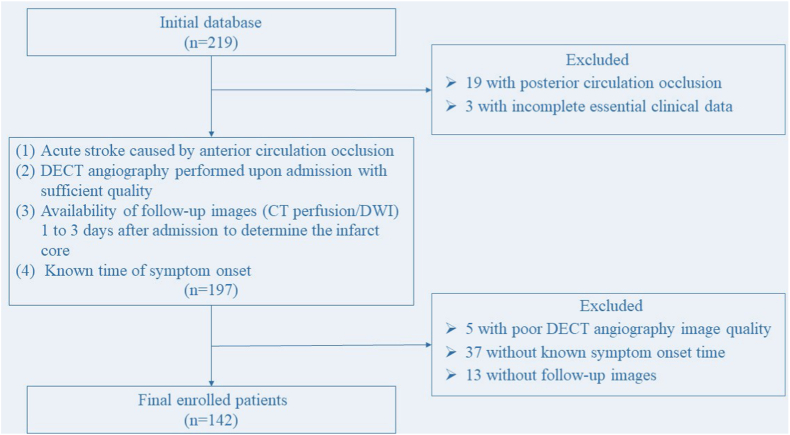
A flowchart of the stroke patient-enrollment process.

The data of acute stroke patients admitted to Shanghai Sixth People's Hospital and Affiliated Hospital of Nantong University from August 2016 to May 2022 were retrospectively analyzed. The study's inclusion criteria as follows [1]: acute stroke caused by anterior circulation occlusion [2]; DECT angiography performed upon admission with sufficient quality (The image quality that enables healthcare professionals to perform image diagnosis and measure the NWU of early infarct) [3]; availability of follow-up images (CT perfusion/DWI) 1–3 days after admission to determine the infarct core; and [4] ascertained time of stroke onset. The study extracted demographic information from medical and follow-up records. This study was reviewed and approved by Shanghai Sixth People's Hospital (No.2020-212). A flowchart of the patient-enrollment process is shown in Fig. 1.

### 2.2 DECT angiography acquisition

Two X-ray tubes were used to capture DECT angiography images using a scanner (Siemens, Germany). The scanners had the following settings: Reformatted section thickness and increment: 1.0 mm and 0.7 mm; reference tube current-time product: for the 90-kVp tube (90 mAs) and Sn150-kVp tube (69 mAs); collimation: 128 × 0.6 mm; rotation time: 0.25 s. Following a bolus injection of 30–40 ml of saline, the recruited patients received contrast agent (Bayer, Germany) into the vein (5 ml/s, 1.5 ml/kg). Software (syngo.via VB20A, Germany) was used to evaluate the DECT angiography data. The VNC and VM (40–140 keV, 20-keV interval) images of the infarcted region were reconstructed derive from contrast-enhanced images.

### 2.3 Acquisition of NWU values

**Fig. 2:**
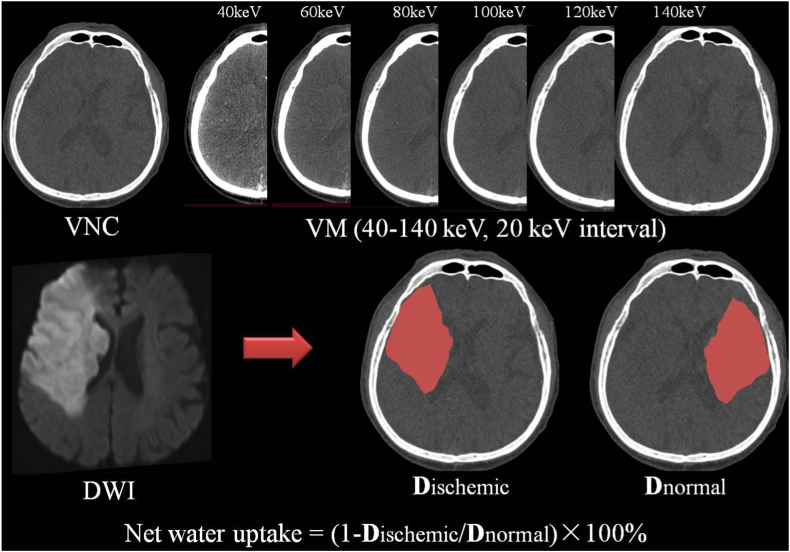
Determination of % net water uptake (NWU) per volume of early infarct on virtual images derived from DECT angiography. The early infarct (i.e., the core lesion) was identified with the reference of DWI. The mean CT density of the infarct core (D*ischemic*) was then calculated in relation to the normal CT density (D*normal*) measured on a mirrored contralateral healthy brain region of interest.

CT density was quantified on virtual images at admission to quantify the NWU in the ischemic brain tissue of stroke patients. CT density was acquired retrospectively by two radiologists with experience in neurovascular imaging more than eight years. DECT angiography identified ischemic brain tissue by referring to the normal area of the contralateral hemisphere, and the follow-up images were used to enhance the precision degree of the ischemic cerebral region of interest (ROI) (Fig. 2). The maximum infarct area of early hypoattenuating infarct on the axial positions of VNC and VM images derived from DECT angiography was assessed using measurements of CT density (D*ischemic*). A mirrored ROI was positioned on the healthy cerebral tissue of the contralateral hemisphere (D*normal*). Both CT density (D*normal* and D*ischemic*) were then utilized for calculating NWU of ischemic cerebral tissue (Equation (1)).

(1) $$\%\text{NWU}=\left(1‐\frac{Dischemic}{Dnormal}\right)\times 100$$

### 2.4 Statistical analysis

Statistical calculations in this study were conducted using SPSS (version 26.0) and MedCalc (version 20.0.2). Patient groups (within or beyond 4.5 h) were compared using the student's t-test, Mann–Whitney *U* test, and the chi-squared test for variables. The inter-reader reproducibility of densitometric measurements on VNC and VM images was assessed using the intraclass correlation coefficient (ICC). A value of ICC >0.75 indicated good agreement. Receiver operating characteristic (ROC) curves were plotted to derive the optimal cut-off values for significant baseline characteristics and NWU values to discriminate stroke patients between within and beyond 4.5 h. The area under the curve (AUC), cut-off value, sensitivity, specificity, accuracy, positive predictive value (PPV), and negative predictive value (NPV) of these parameters were calculated for differential diagnosis. The Spearman rank-order correlation coefficient was applied to assess the relation among significant baseline characteristics, multi-NWU values and stroke onset time. A p-value <0.05 was considered significant.

## 3 Results

Within the study period, 142 patients fulfilled the inclusion criteria with a mean age of 63.97 years (63.97 ± 13.09, range: 27–89). Of these, 78 (54.93 %) and 64 (45.07 %) stroke patients underwent DECT angiography within and beyond 4.5 h, respectively. Baseline characteristics were comparable and summarized in Table 1. The stroke patients within 4.5 h had lower median National Institute of Health stroke scale (NIHSS) score on admission (p-value = 0.005). The ROC curve analysis revealed that the NIHSS score on admission with a cut-off value of 16 yielded an AUC of 0.64 (95 % CI: 0.55–0.71). There were no significant intergroup differences in the other characteristics (p-value >0.05).

**Table 1:** Baseline characteristics of the patients.

Characteristics	within 4.5 h (n = 78)	beyond 4.5 h (n = 64)	F/χ^2^/Z-value	*p*-value
Sex (male, n, %)	56 (71.79)	51 (79.69)	1.18	0.28
Age (years), mean(SD)	67.92 ± 13.85	64.19 ± 13.37	0.10	0.11
Smoking, n (%)	22 (28.21)	16 (25.00)	0.18	0.67
Hypertension, n (%)	60 (76.92)	54 (84.38)	1.23	0.27
Hyperlipidemia, n (%)	3 (3.85)	4 (6.25)	0.43	0.51
Diabetes, n (%)	30 (38.46)	20 (31.25)	0.80	0.37
Atrial fibrillation, n (%)	12 (15.38)	10 (15.63)	0.01	0.97
Coronary artery disease, n (%)	6 (7.69)	8 (12.50)	0.91	0.34
Admission NIHSS, median(IQR)	13 (10–16)	15 (11–18)	−2.77	0.006*
Collateral circulation status			0.349	0.55
0	0 (0)	1 (1.56)		
1	12 (15.38)	10 (15.63)		
2	35 (44.87)	30 (46.87)		
3	31 (39.75)	23 (35.94)		
Onset time(hours), median (IQR)	3.5 (3.0–4.0)	7.0 (5.0–7.5)	−10.33	<0.001*

SD: standard deviation, NIHSS: National Institutes of Health stroke scale, IQR: interquartile range, SD: standard deviation, *: p < 0.05.

The CT density of the ischemic brain area and normal area of the contralateral hemisphere measured on VNC and VM images yielded an excellent inter-observer agreement (ICC values: 0.86–0.99). Stroke patients within 4.5 h had different degrees of significantly lower NWU values within the infarct area on all VNC and VM images compared with the values obtained in those beyond 4.5 h (p-value <0.001) (Table 2).

**Table 2:** The NWU values of the ischemic area were derived from VNC and VM images of patients with stroke occurrence within 4.5 h of and beyond 4.5 h after symptom onset.

NWU values（%）	within 4.5 h (n = 78)	beyond 4.5 h (n = 64)	F-value	*p*-value
VNC，mean ± SD	6.75 ± 4.39	12.17 ± 3.83	3.19	<0.001*
VM(40 keV), mean ± SD	13.51 ± 5.62	21.77 ± 3.44	30.58	<0.001*
VM(60 keV), mean ± SD	14.61 ± 6.51	28.62 ± 6.27	2.17	<0.001*
VM(80 keV), mean ± SD	12.35 ± 7.11	24.31 ± 6.73	0.75	<0.001*
VM(100 keV), mean ± SD	11.33 ± 5.39	19.51 ± 6.64	4.97	<0.001*
VM(120 keV), mean ± SD	9.51 ± 4.85	16.38 ± 5.97	2.41	<0.001*
VM(140 keV), mean ± SD	8.49 ± 4.43	15.17 ± 5.31	1.30	<0.001*

SD: standard deviation, VNC: virtual non-contrast, VM: virtual monoenergetic, *: p < 0.05.

**Fig. 3:**
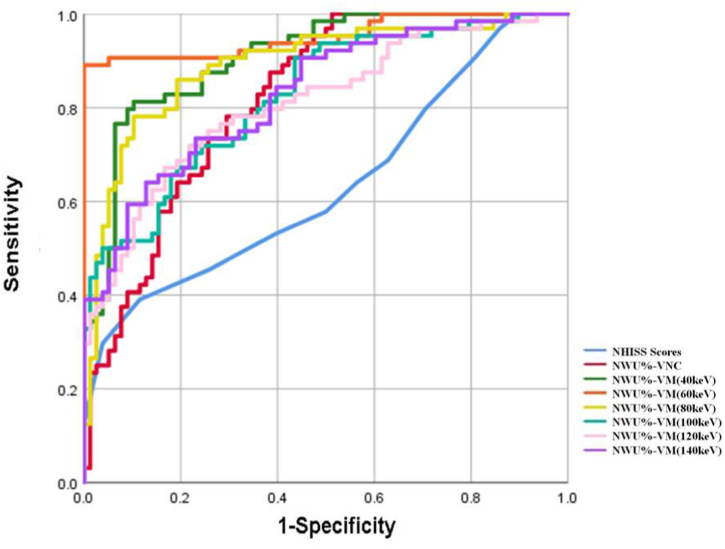
ROC curve of NWU values measured on VNC and VM images for differentiating stroke patients within and beyond 4.5 h. The best diagnostic performance for predicting the stroke patients within 4.5 h could be achieved with an NWU-VM (60 keV) ≤ 24.37 % as the threshold.

The predictive efficiency of NIHSS scores on admission and multi-NWU values derived from different virtual images reconstructed from DECT angiography was shown on Table 3 and Fig. 3. The AUC that discriminated this group of patients from those beyond 4.5 h according to NWU values was in the range of 0.81–0.95. Compared with significant baseline characteristics (indicated by NIHSS scores on admission), NWU values determined using VNC and VM images within the infarct area were all associated with a larger AUC value and better diagnostic performance (p-value <0.05). The highest predictive efficiency was found for NWU values determined using VM (60 keV) images within the infarct area with a cut-off value of 24.37 %, AUC of 0.95 (95 % CI, 0.90–0.98), sensitivity of 100 %, and specificity of 89.06 %, accuracy of 95.07 % (except 40 keV, all p-value< 0.05). The analysis based on ROC curve revealed that the NWU values determined using VM (60 keV) images within the infarct area with a cut-off value of 19.32 % yielded an AUC, sensitivity, specificity, and accuracy of 0.91 (95 % CI, 0.85–0.95), 89.74 %, 81.25 %, and 85.92 %, respectively. A comparison of the diagnostic efficiency of NCCT and CTA revealed that NWU values determined using VM (60 and 40 keV) images within the infarct area were associated with a larger AUC value and superior diagnostic performance compared to the NWU values determined using VNC images (AUC: 0.82 [95 % CI, 0.75–0.88]; sensitivity: 61.54 %; specificity: 87.50 %; and accuracy: 73.24 %) (p-value <0.05).

**Table 3:** The performance of NWU values of ischemic areas derived on VNC and VM images of patients with stroke occurrence within 4.5 h of and beyond 4.5 h after symptom onset.

Characteristics	Youden index	Cutoff value	AUC（95 % CIs）	PPV(%)	NPV(%)	Sensitivity (%)	Specificity (%)	Accuracy (%)
Admission NIHSS*†	0.28	≤16	0.64 (0.55–0.71)	63.91	73.53	88.46	39.06	66.20
NWU%-VNC*	0.49	≤7.96	0.82 (0.75–0.88)	85.68	65.11	61.54	87.50	73.24
NWU%-VM40keV†	0.71	≤19.32	0.91 (0.85–0.95)	85.43	86.68	89.74	81.25	85.92
NWU%-VM60keV†	0.89	≤24.37	0.95 (0.90–0.98)	91.77	100	100	89.06	95.07
NWU%-VM80keV*	0.68	≤20.33	0.89 (0.83–0.94)	83.32	86.17	89.74	78.12	84.51
NWU%-VM100keV*	0.48	≤16.40	0.83 (0.75–0.88)	75.04	74.12	80.77	67.19	76.06
NWU%-VM120keV*	0.51	≤13.86	0.81 (0.74–0.87)	75.58	76.83	83.33	67.19	76.06
NWU%-VM140keV*	0.51	≤13.23	0.83 (0.76–0.89)	74.68	80.43	87.18	64.06	76.76

CI: confidence interval, PPV: positive predictive value, NPV: negative predictive value, NIHSS: National Institutes of Health stroke scale, VNC: virtual non-contrast, VM: virtual monoenergetic, *: There was a difference in diagnostic performance with 60 keV (p < 0.05), †: There was a difference in the diagnostic performance on using VNC images (p < 0.05).

**Fig. 4:**
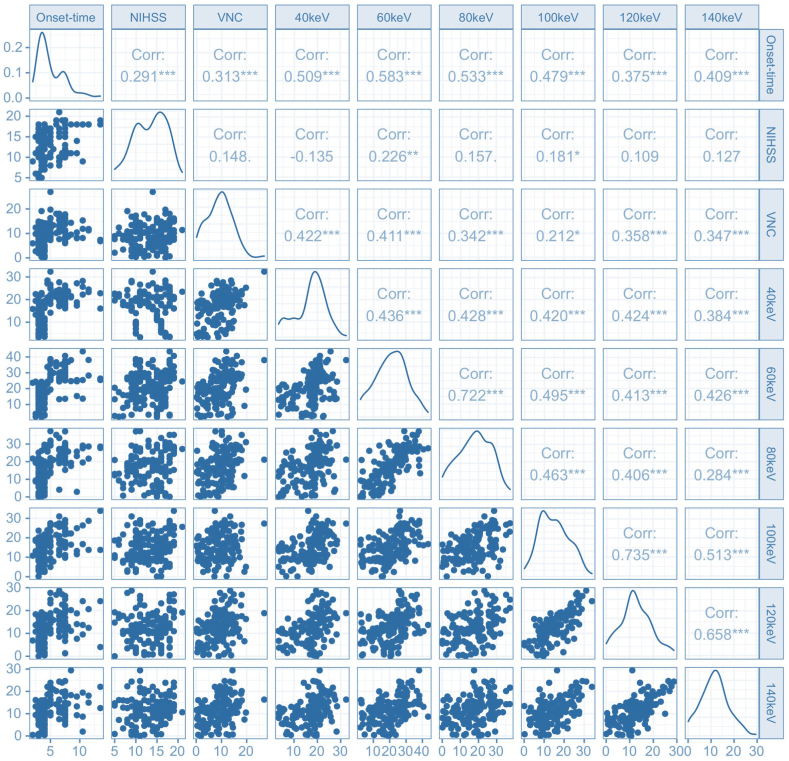
The partial Spearman analysis showed that the NIHSS scores and the NWU values determined using VNC and VM images within the infarct area positively correlated with the stroke onset time (p < 0.001). NWU values measured on VM (60 keV) images displayed the most robust correlation with the stroke onset time (r-value = 0.58, p-value <0.001).

The Spearman analysis showed that NIHSS scores and NWU values determined using VNC and VM images within the infarct area positively correlated with the stroke onset time (p < 0.001). In addition, the NWU values determined using VM (60 keV) images within the infarct area were most strongly associated with the stroke onset time (r-value = 0.58, p-value <0.001), whereas NIHSS scores on admission showed the weakest association (r-value = 0.29, p-value <0.001). Further details about correlation analysis are shown in Fig. 4.

## 4 Discussion

Intravenous thrombolysis is the effective and safe AIS management for the patient with salvageable brain tissue, particularly those stroke onset within 4.5 h [2]. The major challenge in intravenous thrombolysis for stroke patients is to accurately determine the time of stroke onset [9]. Water uptake serves as a dependable indicator of ischemic lesion age in acute stroke, and NWU values determined using conventional NCCT on admission are a quantitative imaging biomarker for ischemic cerebral tissue edema [14,24]. Various studies indicated that the VNC and VM images derived from DECT angiography can highlight early cerebral edema and might increase the sensitivity of the NWU in the ischemic area [25,26]. The primary finding of the study indicates that NWU derived from DECT angiography images is feasible and correlates well with time between the stroke onset of clinical symptoms and imaging; therefore, if suspected, it may be feasible just to perform DECT angiography and skip the NCCT and CTP. Furthermore, the NWU values determined using VM (60 keV) images had the highest predictive efficiency.

The stroke patients within 4.5 h had lower NIHSS scores on admission than those with stroke occurrence beyond 4.5 h (p-value <0.05). The existence of cerebral edema in AIS significantly influences the degree of severity of neurological deficits [27]. Neurological deficit severity scored using the NIHSS is the most important determinant of final functional outcome in AIS patients and scores decrease over time [28]. However, NIHSS score is also associated with other factors [29,30], so NHISS score lacks specificity (only 39.06 %) in predicting the onset time of stroke. There was no statistical difference in collateral circulation status between the patients in two groups. So, we believe that collateral circulation has little impact on our results in this study.

The stroke patients within 4.5 h had lower NWU values on all VNC and VM images derived from DECT angiography than did those with stroke occurrence beyond 4.5 h (p-value <0.001). According to previous reports, DECT angiography can capture different characteristic absorption profiles of substances and could be used to obtain virtual “NCCT” and “CTA” images [19,20]. Furthermore, it showed greater sensitivity in identifying ischemic brain tissue as an area of lower attenuation value compared with conventional CT [21,31,32]. NCCT can always be used to monitor ischemic edema after stroke onset based on detectable attenuation of the decreased density of ischemic brain tissue [33]. NWU values, which directly quantify the degree of hypoattenuation within ischemic brain tissue on NCCT, can be used as a surrogate imaging biomarker for predicting the ischemic lesion age and have been increasingly applied in recent times [13,34]. In addition, CTA-based hypoattenuation is also a method to evaluate ischemic brain damage, as it provides information about cerebral perfusion that cannot be obtained using NCCT alone and seems to be more useful and reliable than NCCT [16,17]. Thus, our results show that the NWU values determined using VNC and VM images derived from DECT angiography could be sensitive and reliable for identifying stroke patients within 4.5 h.

Although all NWU values determined using the VNC and VM images have high predictive efficiency, the best value that could serve as a clinical reference needs to be selected. ROC analysis revealed that NWU values determined using VM (60 keV) images derived from DECT angiography had the highest predictive efficiency (AUC, 0.95; sensitivity, 100 %; specificity, 89.06 %) of all the NWU values measured using VNC and VM images (except 40 keV, all p-value <0.05). In addition, NWU values determined using VM (60 keV) images showed the strongest positive correlation with the stroke onset time. The detection of cerebral edema is based on tissue attenuation values or differences in the water-to-background contrast [35]. VM image reconstruction using DECT scans at enhances the image quality of the head in comparison to the image quality achieved using routine CT [36]. According to a review, iodine noise-optimized resulted in the best image quality at 40–70 keV [37]. Stahl et al. [26] indicated that the energy levels of VM images affect the ability to identify early ischemic brain tissue changes, with VM images at 70 keV exhibiting the optimal diagnostic accuracy. Although our results are not exactly the same as those of previous studies, the keV of reconstruction images is very close. The method we studied is DECT angiography, iodine contrast agent may affect the water-to-background contrast in brain tissue. Furthermore, the different DECT equipment types and scan parameters employed may also lead to differences in the results. In addition, we found that NWU values determined using VM (60 and 40 keV) images afforded a better diagnostic performance than did NWU values measured using VNC images (AUC, 0.82; sensitivity, 61.54 %; specificity, 87.50 %) in our study (p-value <0.05). VNC images obtained from DECT angiography using a specific algorithm were tailored for imaging iodine-related applications and proved a promising alternative to true NCCT images [38]. Our results indicated that NWU values determined using VM images derived from DECT angiography were also better than conventional NCCT in estimating the amount of affected tissue and the time of symptom onset within 4.5 h.

This study has several limitations. First, the study relied on a rather small sample of stroke patients. Further studies with a greater number of patients are necessary to improve the generalizability of the results. Second, the measurement of NWU values depends on the visual hypoattenuation to draw the ROI. In this study, the two experienced radiologists could judge the hypoattenuation in the ischemic area with acceptable objectiveness by using a standardized imaging protocol and follow-up images. Automation methods of drawing the ROIs are also being studied. Third, Patients were recruited from two distinct medical centers, resulting in unavoidable variations in DECT angiography scanning. Both centers employed the same model of DECT equipment, with standardized scanning parameters established prior to the study to minimize their influence. Finally, to minimize radiation exposure, we chose not to conduct an additional NCCT scan However, it has been reported that VNC images are superior to NCCT in identifying acute ischemia due to their ability to preserve edema [22]. Hence, the use of VNC images can be an alternative method that is no worse than true NCCT.

DECT angiography-based quantification of NWU acquired in conjunction with CTP accurately detected stroke within 4.5 h. This method can aid in determining whether intra-arterial treatment should be administered in unknown stroke onset time patients. The NWU values measured on VM images with an energy level of 60 keV could serve as a crucial biomarker for stroke onset time.

## Data availability statement

The authors do not have permission to share data.

## CRediT authorship contribution statement

**Jiang Jingxuan:** Writing – original draft, Methodology, Investigation, Formal analysis, Data curation, Conceptualization. **Guan Baohui:** Writing – original draft, Visualization, Validation, Resources, Methodology, Investigation, Formal analysis, Data curation. **Zhou Jingyi:** Writing – review & editing, Writing – original draft, Validation, Software, Resources, Methodology, Investigation, Formal analysis, Data curation. **Gu Hongmei:** Writing – review & editing, Resources, Methodology, Formal analysis, Data curation. **Li Minda:** Investigation, Formal analysis, Data curation. **Hua Ye:** Methodology, Formal analysis, Data curation. **Li Yuehua:** Writing – review & editing, Validation, Software, Resources, Methodology, Investigation, Funding acquisition, Formal analysis, Data curation, Conceptualization.

## Declaration of competing interest

The authors declare the following financial interests/personal relationships which may be considered as potential competing interests:

Yuehua Li reports financial support was provided by 10.13039/501100001809National Natural Science Foundation of China (Grant No. 8225024 and No. 81871329). Yuehua Li reports financial support was provided by Shanghai Medical Rising Star Talent Fund; Shanghai Science and Technology Innovation Action Plan (No. 20S31907300). If there are other authors, they declare that they have no known competing financial interests or personal relationships that could have appeared to influence the work reported in this paper.
